# microRNA‐19b‐3p‐containing extracellular vesicles derived from macrophages promote the development of atherosclerosis by targeting JAZF1

**DOI:** 10.1111/jcmm.16938

**Published:** 2021-12-14

**Authors:** Qingshan Wang, Yuandi Dong, Haiyang Wang

**Affiliations:** ^1^ Department of Vascular Surgery The First Affiliated Hospital of Harbin Medical University Harbin China; ^2^ Department of Vascular Surgery Heilongjiang Provincial Hospital Harbin China; ^3^ Department of Hepatopancreatobiliary Surgery Harbin Medical University Cancer Hospital Harbin China

**Keywords:** atherosclerosis, extracellular vesicles, JAZF1, macrophages, microRNA‐19b‐3p, vascular smooth muscle cells

## Abstract

Atherosclerosis has been regarded as a major contributor to cardiovascular disease. The role of extracellular vesicles (EVs) in the treatment of atherosclerosis has been increasingly reported. In this study, we set out to investigate the effect of macrophages‐derived EVs (M‐EVs) containing miR‐19b‐3p in the progression of atherosclerosis, with the involvement of JAZF1. Following isolation of EVs from macrophages, the M‐EVs were induced with ox‐low density lipoprotein (LDL) (ox‐LDL‐M‐EVs), and co‐cultured with vascular smooth muscle cells (VSMCs). RT‐qPCR and western blot assay were performed to determine the expression of miR‐19b‐3p and JAZF1 in M‐EVs and in VSMCs. Lentiviral infection was used to overexpress or knock down miR‐19b‐3p. EdU staining and scratch test were conducted to examine VSMC proliferation and migration. Dual‐luciferase gene reporter assay was performed to examine the relationship between miR‐19b‐3p and JAZF1. In order to explore the role of ox‐LDL‐M‐EVs carrying miR‐19b‐3p in atherosclerotic lesions in vivo, a mouse model of atherosclerosis was established through high‐fat diet induction. M‐EVs were internalized by VSMCs. VSMC migration and proliferation were promoted by ox‐LDL‐M‐EVs. miR‐19b‐3p displayed upregulation in ox‐LDL‐M‐EVs. miR‐19b‐3p was transferred by M‐EVs into VSMCs, thereby promoting VSMC migration and proliferation. mir‐19b‐3p targeted JAZF1 to decrease its expression in VSMCs. Atherosclerosis lesions were aggravated by ox‐LDL‐M‐EVs carrying miR‐19b‐3p in ApoE^−/−^ mice. Collectively, this study demonstrates that M‐EVs containing miR‐19b‐3p accelerate migration and promotion of VSMCs through targeting JAZF1, which promotes the development of atherosclerosis.

## 1 INTRODUCTION

Atherosclerosis is regarded as a chronic disorder of the arterial wall, accounting for the poor quality of life and mortality across the globe.^1^ Atherosclerosis is characterized by obstruction of the vascular lumen in the inner layer of blood vessels due to intimate lesions, namely, atheroma or atheromatous plaques, which in severity can abate tunica media.^2^ Atherosclerosis is a multifocal inflammatory reaction, with bacterial and viral infections as potential risk factors.^3^ Moreover, the aberrant proliferation of vascular smooth muscle cells (VSMCs) in arterial walls has been identified a crucial pathogenic factor for atherosclerosis.^4^ In light of the preceding literature, it is imperative to determine the molecular mechanism in the regulation of VSMC proliferation for the treatment of atherosclerosis.

Extracellular vesicles (EVs) are signalling organelles released by various cell types, that are highly conserved in prokaryotes as well as eukaryotes.^5^ An existing study demonstrated the ability of macrophages to secrete EVs, including exosomes, microvesicles, as well as apoptotic bodies.^6^ Intriguingly, the participation of macrophages has been identified in atherosclerosis pathogenesis.^7^ Additionally, EVs have been highlighted as novel therapeutic targets for the treatment of atherosclerosis.^6^ Moreover, EVs by comprising of different DNAs, proteins, mRNAs, as well as microRNAs (miRs) play a vital role in intercellular communications in a variety of diseases.^8^ Notably, miRs have been identified as a family of small (22 nucleotides) noncoding RNAs which can regulate gene expression in a post‐translational manner.^9^ Intriguingly, an existing study identified the involvement of miRs in the pathogenesis of atherosclerosis.^10^ On the basis of the bioinformatics results obtained in the current study, miR‐19b‐3p was differentially expressed in macrophages‐derived EVs (M‐EVs). As previously reported, miR‐19b by inducing dysfunction of endothelial cells can exacerbate the development of atherosclerosis.^11^ Moreover, EVs containing miR‐19b‐3p might be associated with PM‐related cardiovascular disease such as atherosclerosis.^12^ Based on the bioinformatics analysis, zinc finger gene 1 (JAZF1) was identified as a target of miR‐19b‐3p. The transcription factor JAZF1 is regarded as a type of zinc finger protein binding to the nuclear orphan receptor TR4.^13^ It has been reported that upregulated JAZF1 could protect ApoE^−/−^ mice against atherosclerosis through inhibition of hepatic cholesterol synthesis in a CREB‐dependent manner.^14^ On the basis of the aforementioned literature, the current study proposed that M‐EVs containing miR‐19b‐3p may affect the progression of atherosclerosis by regulation of JAZF1.

## 2 MATERIALS AND METHODS

### 2.1 Ethical approval

This study was conducted with the approval of the ethics committee of The First Affiliated Hospital of Harbin Medical University. Great efforts were made to minimize the suffering of the experimental animals used in the study.

### 2.2 Cell treatment

The RAW264.7 macrophage line provided by American Type Culture Collection (ATCC, VA, USA) was cultured in Dulbecco's modified Eagles Medium (DMEM) containing a combination of 10% (v/v) EVs‐depleted fetal bovine serum (FBS), 1% (v/v) P‐S and 1 mM sodium pyruvate. The mouse arterial VSMC cell line purchased from Beijing Solarbio Science & Technology Co., Ltd. was cultured in DMEM containing 10% (v/v) FBS and 1% (v/v) P‐S.

The original culture medium was discarded, after which the cells were detached with 0.25% trypsin solution. Then, culture medium was added to terminate the digestion, and the adherent cells were transferred into suspension using a pipette and then transferred into other bottles containing culture medium for subsequent culture.

Macrophages were used as negative control (NC), or treated with miR‐19b‐3p mimic or miR‐19b‐3p KD. Lentiviral overexpression vectors and interference plasmid vectors (Invitrogen) were subsequently constructed. During transfection, the macrophages (5.0 × 10^7^ cells/ml) were seeded into a six‐well plate with 2 ml per well. Upon attain 50% cell confluency, the cells were incubated with the prepared lentivirus supernatant (concentration higher than 10^7^ TU/ml). After 24 h of infection, the solution was renewed with a complete medium. Upon attaining 80% confluency, the complete medium was renewed with serum‐free medium for a regimen of incubation for 24 h. Next, the EVs were separated by differential centrifugation. The expression of miR‐19b‐3p in the isolated EVs was determined by reverse transcription‐quantitative polymerase chain reaction (RT‐qPCR); the results of which were compared with those of the uninfected macrophages.

### 2.3 Isolation and purification of EVs

Macrophages in each group were cultured in serum without EVs. Upon attaining 70%–80% macrophage confluency, the medium was centrifuged for 10 min at 300 *g* to isolate the supernatant. Next, the supernatant was centrifuged at low speed (2000 *g*, 10 min) to remove any cell debris. Subsequently, the supernatant was centrifuged at 10,000 *g* for 30 min, and then ultracentrifuged at 100,000 *g* for 120 min (Optima L‐100XP, Beckman Coulter Inc.). Finally, the outer coating body was washed twice with an abundant amount of phosphate‐buffered saline (PBS), followed by centrifugation for 120 min at 100,000 *g*. The EVs underwent isolation and purification by sucrose density gradient. The isolated EVs were re‐suspended in 0.25 M sucrose and loaded with sucrose layers of 2, 1.3, 1.16, 0.8 and 0.5 M in gradient, followed by centrifugation at 100,000 *g* through ultracentrifugation for 2.5 h. The components with different densities were extracted from the brim of the sample tube and analysed by sodium dodecyl sulphate‐polyacrylamide gel electrophoresis separation (SDS‐PAGE). The suspension containing EVs was rinsed twice with an abundant volume of PBS, and centrifuged at 100,000 *g* at 4°C for 120 min. The concentrated EVs were preserved at −80°C for subsequent experimentation.

### 2.4 Transmission electron microscopy (TEM)

The EVs derived from the macrophages were subjected to co‐culture with VSMCs and purification. The EVs were fixed using 5% glutaraldehyde (in the dark) in 0.1 M phosphate buffer at 4°C. The EVs (20–40 μm) separated from the re‐suspended droplets were placed on a copper mesh specifically used for electron microscopy (Hitachi H‐7650), followed by counter‐staining with 20 μl of 2% phosphotungstic acid for 10 min. All samples were analysed under electron microscopy at 100 KV.

### 2.5 Western blot assay

The surface markers tumour susceptibility gene 101 (TSG101), CD9, CD63 and endoplasmic reticulum protein marker calenxin were identified by western blot assay. The suspension of EVs was concentrated, after which the protein content was determined using BCA Kits (23227, Thermo Fisher Scientific Inc.). SDS‐PAGE gel was prepared and protein denaturation and electrophoresis were performed, after which the protein was transferred onto a membrane and the concentration of cell marker protein was determined. Glyceraldehyde‐3‐phosphate dehydrogenase (GAPDH) antibody was regarded as an internal reference. The relative content of the target protein was expressed as the gray value of the target protein band to that of the internal reference protein band. The key antibodies were as follows: CD63 (1: 1000, ab216130, Abcam Inc.), CD9 (1: 1000, ab223052, Abcam), TSG101 (1: 1000, ab125011, Abcam), calnexin (1: 1000, ab22595, Abcam), immunoglobulin G (IgG) (1: 5000, ab6721, Abcam), GAPDH (1: 2500, ab9485, Abcam). Image J software was used to quantify the gray level of each band in the western blot images.

### 2.6 Nanoparticle tracking analysis (NTA)

A total of 20 μg EVs were dissolved in 1 ml PBS for 1 min with uniform distribution of EVs. Next, the particle size distribution of EVs was directly observed and measured using a NanoSight nanoparticle tracking analyzer (Malvern Instruments Co., Ltd.).

NanoSight (NTA 3.2 Dev Build 3.2.16, Merkel Technologies) was adopted to perform NTA for characterization of the concentrations of M‐EVs and ox‐low density lipoprotein (LDL)‐induced M‐EVs (ox‐LDL‐M‐EVs). The size distribution and concentration of vesicles were measured by dynamic light scattering (DLS) using the Zetasizernano ZS90 analysis system (Zetasizer 7.12, Malvern instruments). The particle size distribution map was established, where the *X*‐axis was indicative of the distribution of estimated particle size (nm), and the *Y*‐axis was indicative of the relative percentage. The particle size distribution was plotted based on the estimated particle size (nm) on the *X*‐axis and the concentration (particles/ml) on the *Y*‐axis. Additionally, the particle size distribution diagram based on the intensity was plotted in strict accordance with the particle size (nm) on the *X*‐axis and the intensity (a.u.) on the *Y*‐axis.

### 2.7 Tracing experiment of VSMCs‐internalized EVs

VSMCs and internalized EVs were labelled using the membrane dye PKH67 (Invitrogen) and were rinsed and re‐suspended in serum‐free medium. Next, VSMCs were seeded on a single‐layer glass chassis (Cellvis) and co‐cultured with the PKH67‐labelled vesicles for periods of 0, 1, 6 and 24 h, followed by three rinses with PBS, fixation in 4% paraformaldehyde, staining with DiI and ultimately observation under confocal microscopy (Leica TCS Sp8).

### 2.8 3‐(4,5‐dimethylthiazol‐2‐yl)‐2,5‐diphenyl tetrazolium bromide (MTT) assay and 5‐ethynyl‐2′‐deoxyuridine (EdU) staining

The effect of M‐EVs on cell proliferation was examined by cell counting and EdU staining (Riobio). Briefly, a concentration of 2 × 10^3^ cells/well (4 replicates in each group) were seeded into a 96‐well plate and cultured in a medium containing miR‐19b‐3p inhibitor with or without M‐EVs (8 μg/well). M‐EVs were used as control. Subsequently, 10 nm EdU was added for incubation for 12 h. The cells were fixed in 4% paraformaldehyde and stained in strict accordance with the provided manufacturer's instructions. Cell proliferation was observed under fluorescence microscopy.

### 2.9 Scratch test

In the scratch test, 3 × 10^5^ cells/well (three replicates in each group) were placed in a 12‐well plate and for growth until confluency. The monolayer was scratched with the tip of the pipette and rinsed with serum‐free medium to eliminate the isolated cells. Subsequently, the cells were cultured in EVs‐deficient medium containing miR‐19b‐3p inhibitors with or without M‐EVs (80 μg/well), where M‐EVs were used as control. The VSMCs were photographed at 0, 12 and 24 h after injury. Wound closure was calculated as follows: migration area (%) = (*A*_0_ – *A*_n_)/*A*_0_ × 100, where *A*_0_ represents the initial wound area and *A*_n_ represents the wound area at the time of measurement.

### 2.10 miR sequencing

The miRNA microarray data of the EVs were retrieved from the GEO database (GSE132646 and GSE114318). GSE132646 included 12 samples (six control groups and six experimental groups), while GSE114318 included nine samples (six control groups and three experimental groups). The ‘limma’ package of R language was used to analyse the differential expression of several genes. DEmiRNAs were obtained with logFC > 1, *p* < 0.05 as screening threshold. R language ‘ggplot2’ software package was used to plot the volcano map, and the intersection of DEmiRNAs was processed.

Initially, the total RNA content was isolated from control EVs and ox‐LDL‐EVs samples using Trizol reagent (Takara). The total RNA content was separated on 15% three boric acid ethylene diamine tetraacetic acid (EDTA) (TBE) polyacrylamide gel (Invitrogen), and a strip corresponding to miR (18–30 nt) was removed. Next, the miR was reversely transcribed into complementary DNA (cDNA) and amplified. The miRs were sequenced using Illuminutesa HiSeq^TM^ 2000. After that, Novoalign software (v2.07.11) was adopted to process the sequencing results. Next, the paired *t*‐test with double‐sided distribution was adopted to identify the difference of each miR in the M‐EVs and M‐EVs samples, and used Bonferroni method to correct multiple determination. Parts per million (CPM) was used for calculation. Fold changes of each miR (mean CPM of each miR in control EVs/CPM of each miR in ox‐LDL‐EVs) and *p* values were calculated. The *p* value was used to calculate the error detection rate (FDR) of each miR, and was further used as a functional filter to identify significant miRs with fold change ≥2 or ≤0.05 and FDR <0.05. The MEV software was utilized to analyse the expression data.

### 2.11 RT‐qPCR

Total RNA was extracted from the cell lines and frozen tissue samples using the Trizol reagent (15596‐018, Solabio) in strict accordance with the provided instructions. To measure the mRNA expression, PrimeScript™ RT‐PCR kits (Takara) were used to synthesize cDNA from the total RNA content. To determine the miR expression, PrimeScript™ miRNA RT‐PCR kits (b532451, SANGON) were used for reverse transcription following the provided instructions. SYBR Premix Ex Taq^TM^ (Takara) was used for real‐time RT‐qPCR on LightCycler 480 system (Roche Diagnostics GmbH). With β‐actin and U6 serving as internal references, the mRNA and miR expression was standardized. The primers for amplification were designed and purchased by General Biotechnology Co., Ltd. The primer sequences are shown in Table S1. The relative quantitative method (2^−△△Ct^ method) was employed for calculating the relative expression of the target genes.

### 2.12 Dual‐luciferase reporter gene assay

The targeting relationship between miR‐19b‐3p and JAZF1 was verified by a combination of the biological prediction website and the luciferase reporter method. The binding sites between miR‐19b‐3p and JAZF1 were analysed, and the fragment sequences containing the action site were identified. The JAZF1 3’‐UTR sequence containing predicted that the miR‐19b‐3p binding site was inserted into the pGL3 basic vector (Promega) for the synthesis of the firefly/Renilla luciferase report vector pGL3‐basic‐ JAZF1‐3’‐UTR‐wild type (WT); the mutation was pGL3‐basic‐JAZF1‐3’‐UTR‐mutation type (MUT). Next, the HEK‐293 cells were seeded into a 24‐well plate and incubated for 24 h to attain 50%–60% cell confluency. Subsequently, Lipofectamine 2000 was used to co‐transfect with NC‐mimic/miR‐19b‐3p mimic and pGL3‐basic‐ JAZF1‐3’‐UTR‐WT, or NC‐mimic/miR‐19b‐3p mimic and pGL3‐basic‐ JAZF1‐3’ ‐UTR‐MUT into the cells. In addition, all groups were transfected with 10 ng of PRL TK Renilla luciferase. After 24 h of transfection, the cell lysate was collected to measure the luciferase activity. According to the provided manufacturer's instructions, the relative luciferase activity was determined using the dual‐luciferase reporter gene assay system (E1910; Promega) and normalized to Renilla luciferase activity.

### 2.13 Animal model establishment

Male C57BL/6 WT mice and ApoE^−/−^ mice (C57BL/6) were purchased from Beijing Vital River Laboratory Animal Technology Co., Ltd. and housed in specific‐pathogen‐free environment. ApoE^−/−^ mice were fed with a normal diet (ND), high‐fat diet (HFD) (21% crude fat, 0.15% cholesterol, 19.5% casein) depending on the experimental group. The feeding period spanned over 12 weeks, with ad libitum access to food and water. During the experiment, several parameters such as the body weight, blood glucose, cholesterol, LDL, high‐density lipoprotein (HDL), triglyceride and uric acid in serum were measured. After 12 weeks of feeding, the mice fed with ND were injected with PBS solution as control; the mice fed with HFD were injected with PBS solution as control, or injected with ox‐LDL‐M‐EVs or ox‐LDL‐M‐EVs‐miR‐19b‐3p‐KD [10 μg ox‐LDL‐M‐EVs labelled with PKH26 (red)] (each *n* = 10). In order to validate whether the transfection was successful, immunofluorescence was performed 24 h after injection. Subsequently, all the mice were euthanized for molecular biology testing.

### 2.14 Histological staining

After 12 weeks, the intact aorta was carefully separated and divided into 5‐mm frozen sections. Haematoxylin and eosin (HE) staining was conducted to observe the concentration of atherosclerotic lesions of the aortic root. The lipid deposition was determined by measuring the oil red O staining area. The protein expression and distribution of JAZF1 were determined by immunohistochemistry. Paraffin sections (4 μm in thickness) of tissues were collected and dewaxed to water. The antibody used was JAZF1 (1: 500, sc‐376503, Santa Cruz Biotechnology, Inc). The criteria for staining results were set as follows: five lesions were randomly selected under a microscope (200 times) and the number of positive cells in the cells was counted. The density of positive cells could also be semi‐quantitatively graded according to the percentage of positive cells: the number of positive cells <15% was defined as negative (−), 15%–25% as (+ +), 25% ~50% as (+ +), 50%–75% as (+ + +) and >75% as (+ + + +). In the negative group, the number of positive cells was less than 25%; in the positive group, the number of positive cells was more than 25%.

### 2.15 Immunofluorescence

Macrophages, VSMCs and EVs grew into monolayers with confluency on a cell culture glass coverslip. The cells were rinsed with cold PBS, fixed with paraformaldehyde, and then permeated in Tris buffer saline. The cells were sealed at 4°C overnight along with the required primary and secondary antibodies. The main antibody used for macrophages was CD68 (1:1000, ab213363, Abcam), for VSMCs was α‐smooth muscle actin (α‐SMA) (1:200, 55135‐1‐AP, Proteintech Group) and for EVs was CD9 (1:1000, ab223052, Abcam); the secondary antibody was IgG (1:5000, ab6721). Fluorescence images of cells were documented under an inverted Leica fluorescence microscope (IX71) or Leica SP‐8 confocal microscope system. U6 probe and a scrambled sequence were used as positive and negative controls respectively.

### 2.16 Statistical analysis

SPSS 21.0 (SPSS, IBM) was used to analyse the experiment data. The measurement data, from three independent experiments were expressed as mean ± standard deviation. An unpaired *t*‐test was adopted for comparing data between two groups. One‐way analysis of variance (ANOVA) was conducted for comparing data between multiple groups, followed by Tukey's post hoc test. In all statistical references, a value of *p* < 0.05 was indicative of a statistically significant difference.

## 3 RESULTS

### 3.1 M‐EVs are internalized by VSMCs

**FIGURE 1:**
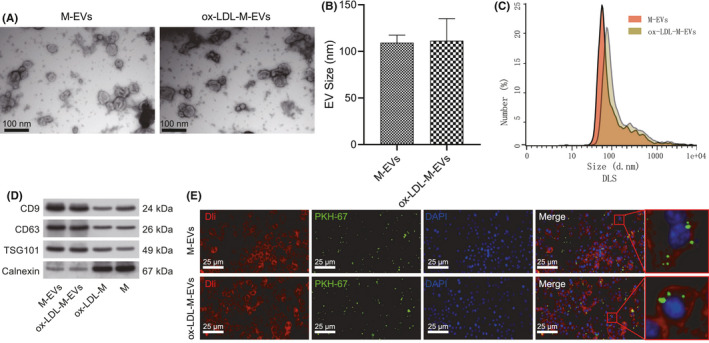
VSMCs internalize M‐EVs. (A) Electron microscopic photos of M‐EVs and ox‐LDL‐M‐EVs. (B) Average particle size of M‐EVs and ox‐LDL‐M‐EVs. *p < 0.05. (C) Particle size distribution of M‐EVs and ox‐LDL‐M‐EVs. (D) The expression of TSG101, CD9, CD63 and calnexin in macrophages and EVs as determined by western blot assay. (E) The internalization of EVs by VSMCs observed by immunofluorescence. Macrophages (M), ox‐LDL‐induced macrophages (ox‐LDL‐M), macrophage‐separated EVs (M‐EVs), and ox‐LDL‐treated macrophage‐separated EVs (ox‐LDL‐M‐EVs). The measurement data were expressed as mean ± standard deviation. Unpaired *t*‐test was used for comparing data between two groups

The RAW264.7 macrophages were cultured with EV‐depleted medium, treated with ox‐LDL or without ox‐LDL (50 μg/ml) for 48 h, after which the supernatant was isolated for separation and purification of EVs. The untreated M‐EVs and ox‐LDL‐M‐EVs were observed under TEM. The results revealed that the EVs were round or cup‐shaped, and ranged in size between 30 and 150 nm (Figure 1A). Nanosight analysis showed that the average size of M‐EVs and ox‐LDL‐M‐EVs were 109.30 and 111.40 nm, respectively, which were within the typical size ranges of EVs (Figure 1B). DLS measurements revealed that the diameters of these particles ranged between 80 and 120 nm (Figure 1C). The results of western blot assay validated that these experimental particles were EVs, and the EVs markers TSG101, CD9 and CD63 were not expressed or rarely expressed in macrophages or ox‐LDL‐M, but were highly expressed in M‐EVs and ox‐LDL‐M‐EVs; the expression of endoplasmic reticulum protein calnexin was limited to macrophages and ox‐LDL‐M, instead of M‐EVs or ox‐LDL‐M‐EVs (Figure 1D). We also used ELISA to detect the content of ox‐LDL in the isolated ox‐LDL‐M‐EVs, and observed that no trace of ox‐LDL in the isolated ox‐LDL‐M‐EVs (Figure S1), thus validating that the isolated EVs were sterile for extensive experimentation. Next, the VSMCs were incubated with the isolated EVs. The EVs were labelled with PKH67 (green fluorescence), the nucleus of VSMCs with 4,6‐diamidino‐2‐phenylin‐dole (DAPI) (blue fluorescence) and the cell membrane with Dil (red fluorescence). The results of fluorescence microscopy revealed that the EVs predominantly surrounded the nucleus or were aligned on the inner surface of the cell membrane after penetrance in the cells, speculating endocytosis as the chief mechanism for internalization of EVs by VSMCs (Figure 1E). These results suggest that VSMCs can essentially internalize M‐EVs.

### 3.2 ox‐LDL‐M‐EVs promote migration and proliferation of VSMCs

**FIGURE 2:**
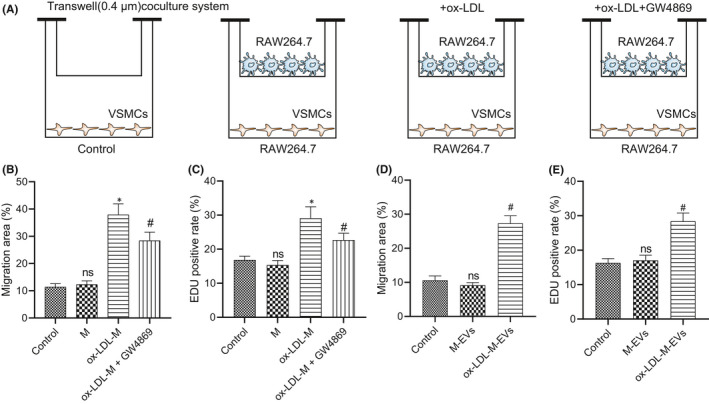
ox‐LDL‐M‐EVs promote migration and proliferation of VSMCs. (A) The co‐culture system of VSMC and macrophages. (B) Quantitative analysis of the effect of macrophages on VSMC migration after different treatment as examined by scratch test. (C) Quantitative analysis of the effect of macrophages on VSMC proliferation after different treatment as examined by EdU staining. (D) Quantitative analysis of the effect of EVs on the migration ability of VSMCs as examined by scratch test. (E) Quantitative analysis of the effect of EVs on VSMC proliferation as examined by EdU staining. *p < 0.05 versus control. ^#^ *p* < 0.05 versus ox‐LDL‐M or M‐EVs. NS indicates no significant difference. The measurement data were expressed as mean ± standard deviation

Next, we determined the effect of ox‐LDL‐M‐EVs on the migration and proliferation of VSMCs. The co‐culture system of VSMCs and macrophages is shown in Figure 2A. The results of EdU staining and scratch test showed that the migration and proliferation of VSMCs were increased significantly after incubation with ox‐LDL‐M (Figure 2B,C); however, the migration and proliferation of VSMCs were markedly decreased by GW4869, an inhibitor for the secretion of EVs. Therefore, we speculated that ox‐LDL‐M can facilitate VSMC migration and proliferation. To further confirm the effect of ox‐LDL‐M‐EVs on VSMCs, we conducted EdU staining and scratch test again to verify whether the EVs stimulate the migration and proliferation of VSMCs. The results showed that the 24‐h migration area of VSMCs carrying ox‐LDL‐M‐EVs was 27% (Figure 2D), with a proliferation rate of 28% as determined measured by EdU staining (Figure 2E). These results demonstrate that ox‐LDL‐M‐EVs can stimulate VSMCs migration and proliferation.

### 3.3 miR‐19b‐3p is upregulated in ox‐LDL‐M‐EVs

**FIGURE 3:**
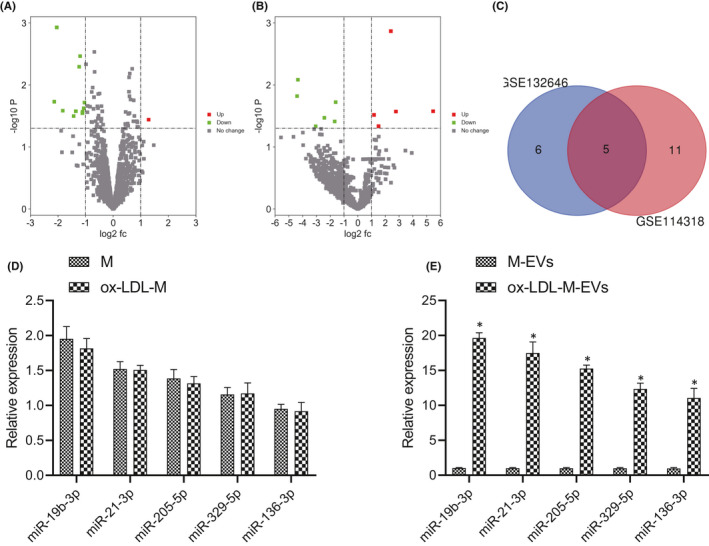
miR‐19b‐3p is upregulated in ox‐LDL‐M‐EVs. (A) Differentially expressed miRNAs in GSE132646. (B) Differentially expressed miRNAs in GSE114318. Red represents upregulated genes and green represents downregulated genes, the gray dot indicates that there is no significant difference, and the horizontal axis indicates the logarithm of 2 of the multiple of difference (FC) between different groups, that is log2 (FC). (C) Venn diagram of intersection from GSE132646 and GSE114318 datasets. (D) The expression of miR‐19b‐3p, miR‐21‐3p, miR‐205‐5p, miR‐329‐5p and miR‐136‐3p in macrophages and EVs. (E) The expression of miR‐19b‐3p, miR‐21‐3p, miR‐205‐5p, miR‐329‐5p and miR‐136‐3p in M‐EVs and ox‐LDL‐M‐EVs. The measurement data were expressed as mean ± standard deviation. Unpaired *t*‐test was used for comparing data between two groups

To identify the potential molecular targets for ox‐LDL‐M‐EVs‐mediated VSMC migration and proliferation, we searched and analysed the miRNA microarray data (GSE132646 and GSE114318) of EVs was analysed from the GEO database. As shown in Figure 3A,B, 12 DEmiRNAs (1 upregulated and 11 downregulated) and 11 DEmiRNAs (five upregulated and six downregulated) were identified respectively. After the intersection of the two groups of DEmiRNAs, five mutual miRNAs were identified (Figure 3C). Among the five miRNAs, the expression of miR‐19b‐3p changed most significantly, so miR‐19b‐3p was chosen for subsequent experimentation.

To verify the reliability of the miR sequencing results, RT‐qPCR was conducted to determine the expression pattern of five miRs (miR‐19b‐3p, miR‐21‐3p, miR‐205‐5p, miR‐329‐5p and miR‐136‐3p) in macrophages containing EV‐depleted supernatant and EVs. The results showed that the expression patterns of the five miRs in macrophages containing EV‐depleted supernatant and ox‐LDL‐M were equivalent, with an exception of significantly higher expression patterns of the five miRs in ox‐LDL‐M‐EVs relative to M‐EVs, while miR‐19b‐3p expression upregulated most significantly (Figure 3D,E). Overall, we identified upregulation of miR‐19b‐3p in ox‐LDL‐M‐EVs.

### 3.4 M‐EVs promotes VSMC migration and proliferation by transferring miR‐19b‐3p

**FIGURE 4:**
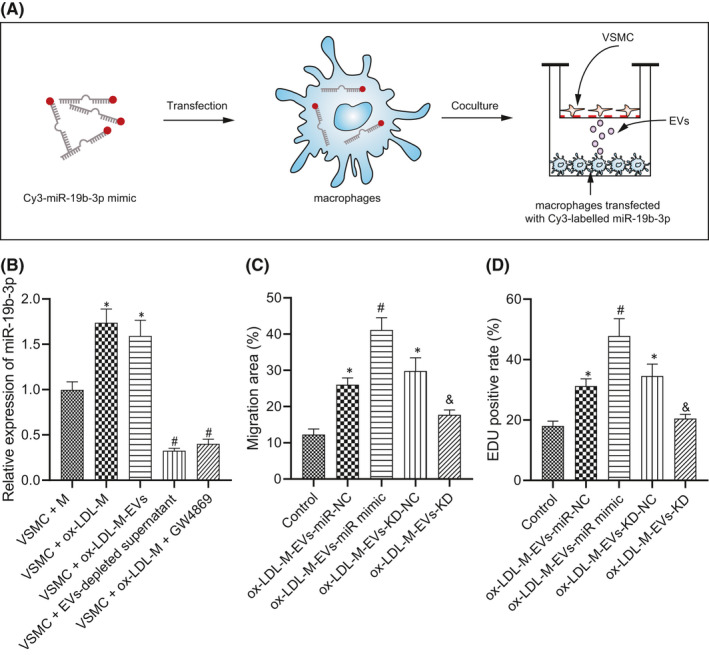
M‐EVs promote VSMC migration and proliferation by transferring miR‐19b‐3p. (A) Schematic diagram of co‐culture of macrophages with VSMCs after transfection with Cy3‐labeled miR‐19b‐3p (red). (B) The relative expression of miR‐19b‐3p after different treatment as determined by RT‐qPCR *p < 0.05 versus VSMCs + M. ^#^ *p* < 0.05 versus VSMC + ox‐LDL‐M or VSMC + ox‐LDL‐M‐EVs. (C) Quantitative analysis of migration ability of VSMCs as examined by scratch test. (D) Quantitative analysis of the VSMC proliferation as examined by EdU staining. *p < 0.05 versus control. ^#^ *p* < 0.05 versus ox‐LDL‐M‐EVs‐miR‐NC. ^&^ *p* < 0.05 versus ox‐LDL‐M‐EVs‐KD‐NC. The measurement data were expressed as mean ± standard deviation. One‐way ANOVA was conducted for comparing data between multiple groups, followed by Tukey's post hoc test

To determine the mechanism by which M‐EVs deliver miR‐19b‐3p into VSMCs, we used Cy3 labelling (red fluorescence) to track the transport of miR‐19b‐3p between cells, and used the Transwell co‐culture device to further investigate the transfer mechanism (Figure 4A). RT‐qPCR results demonstrated that the expression pattern of miR‐19b‐3p was increased in VSMCs co‐cultured with ox‐LDL‐M or ox‐LDL‐M‐EVs, while its expression pattern was reduced upon treatment with an inhibitor of secretion of EVs, GW4869 in VSMCs co‐cultured with ox‐LDL‐M (Figure 4B). The preceding data is indicative for chief transfer of miR‐19b‐3p in the form of EVs.

To further verify our conjecture, we infected miR‐19b‐3p mimic or miR‐19b‐3p‐KD into ox‐LDL‐M‐EVs, followed by incubation of VSMCs; after which the alterations in cell migration and proliferation were determined in each group. After treatment with miR‐19b‐3p mimic, the migration and proliferation of VSMCs were significantly increased (Figure 4C,D). Moreover, the migration and proliferation of VSMCs decreased after treatment with miR‐19b‐3p‐KD, which suggested that the knockdown of miR‐19b‐3p could weaken the stimulative effect of ox‐LDL‐M‐EVs on VSMC migration and proliferation. To exclude the effect of cell proliferation and diffusion in cell migration, transwell assay was conducted, which provided identical results as the scratch test (Figure S2). These results suggest that M‐EVs can stimulate VSMC migration and proliferation by transferring miR‐19b‐3p.

### 3.5 M‐EVs carrying miR‐19b‐3p promote VSMC migration and proliferation by targeting JAZF1

**FIGURE 5:**
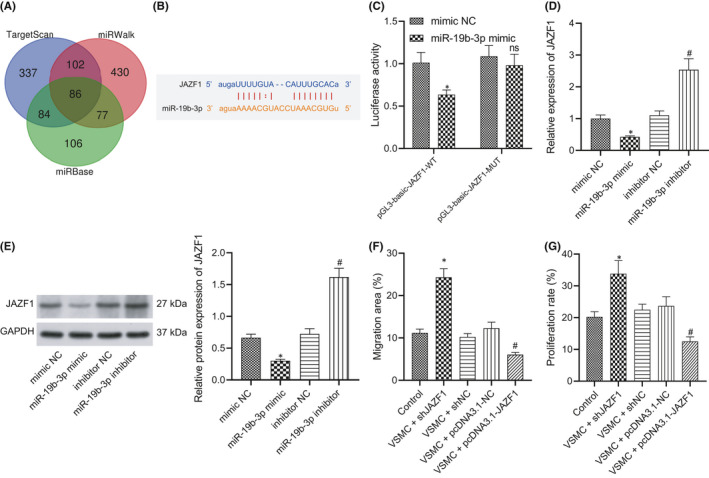
M‐EVs carrying miR‐19b‐3p promote VSMC migration and proliferation by targeting JAZF1. (A) Venn diagram of intersection from TargetScan, miRWalk and miRBase databases. (B) The binding site of miR‐19b‐3p with JAZF1 3’UTR as predicted on TargetScan. (C) Dual‐luciferase gene reporter assay examining whether miR‐19b‐3p targets JAZF1. *p < 0.05 versus mimic NC. NS indicates no significant difference. (D) The change of JAZF1 mRNA expression after addition of miR‐19b‐3p mimic or inhibitor. *p < 0.05 versus mimic NC. ^#^ *p* < 0.05 versus inhibitor NC. (E) The effect of miR‐19b‐3p mimic or inhibitor on the protein expression of JAZF1 as determined by western blot assay. (F) Quantitative analysis of the effect of JAZF1 on the migration ability of VSMCs as observed by scratch test. (G) Quantitative analysis of the effect of JAZF1 on VSMC proliferation by as observed EdU staining. *p < 0.05 versus control or mimic NC. ^#^ *p* < 0.05 versus VSMCs + pcDNA3.1‐NC or inhibitor NC. The measurement data were expressed as mean ± standard deviation. Unpaired *t*‐test was used for comparing data between two groups. One‐way ANOVA was conducted for comparing data between multiple groups, followed by Tukey's post hoc test

In order to elucidate the molecular mechanism of miR‐19b‐3p involved in VSMC migration and proliferation, a combination of TargetScan, miRWalk and miRBase was adopted to predict the target gene, which identified JAZF1 as one of the downstream target genes of miR‐19b‐3p (Figure 5A) and the presence of a binding site between JAZF1 and miR‐19b‐3p (Figure 5B). In order to verify whether JAZF1 is the target gene of miR‐19b‐3p, dual‐luciferase reporter gene assay was adopted to analyse and determine the binding site between miR‐19b‐3p and JAZF1. The results showed that miR‐19b‐3p could directly target 3'UTR of JAZF1 (Figure 5C). To further determine the regulatory effect of miR‐19b‐3p on VSMCs, the VSMCs were treated with miR‐19b‐3p mimic, mimic NC, miR‐19b‐3p inhibitor or inhibitor NC, followed by comparison of the expression pattern of JAZF1 at the mRNA and protein levels. After overexpression of miR‐19b‐3p (miR‐19b‐3p mimic), the expression pattern of JAZF1 was lowered, while it was increased in response to knockdown of miR‐19b‐3p (miR‐19b‐3p‐kd) was increased (Figure 5D,E). These results demonstrated that miR‐19‐3p can inhibit the mRNA and protein levels of JAZF1. To study the role of JAZF1 in VSMC migration and proliferation, specific shRNA was used to selectively knock out JAZF1 or transfect JAZF1 pcDNA3.1 plasmid. Scratch test and EdU staining were conducted to study the migration and proliferation of VSMCs with different treatment protocols (control VSMCs, VSMCs + shJAZF1, VSMCs + SHNC, VSMCs + pcDNA3.1‐NC and VSMCs + pcDNA3.1‐JAZF1). The results revealed that after transfection with sh‐JAZF1, the migration and proliferation of VSMCs were notably enhanced, while opposite trends were observed after JAZF1 overexpression (Figure 5F,G), suggesting the involvement of JAZF1 in the inhibition of VSMC migration and proliferation. As shown in Figure S3, western blot assay demonstrated that ox‐LDL‐M‐EVs induced a decrease in JAFZ1 expression pattern. Overexpression of miR‐19b‐3p further reduced JAFZ1 expression pattern, while knockdown of miR‐19b‐ 3p can restore the expression pattern of JAFZ1. In conclusion, miR‐19b‐3p by specifically binding to JAZF1 can negatively regulate the expression pattern of JAZF1, and facilitate the migration and proliferation of VSMCs by inhibiting JAZF1.

### 3.6 ox‐LDL‐M‐EVs carrying miR‐19b‐3p aggravate atherosclerosis in ApoE^−/−^ mice

**FIGURE 6:**
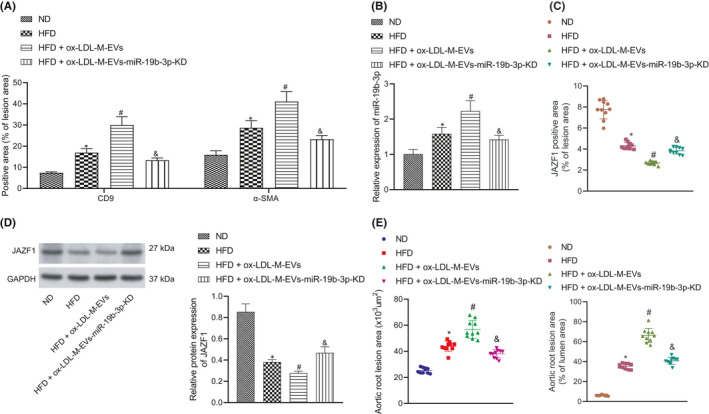
ox‐LDL‐M‐EVs carrying miR‐19b‐3p aggravate atherosclerosis in ApoE^−/−^ mice. (A) The expression of α‐SMA and CD9 in mouse aorta as measured by immunofluorescence. (B) The expression of miR‐19b‐3p as determined by RT‐qPCR. (C) Quantitative analysis results of JAZF1 expression in aorta of mice with different treatment as measured by immunohistochemistry (*n* = 10). *p < 0.05. (D) The protein expression of JAZF1 in aortas of ApoE^−/−^ mice as determined by western blot assay. (E) Quantitative analysis results of HE and oil red O staining for aortic plaque area and lipid deposition, lesion size and lumen area percentage in ApoE^−/−^ mice (*n* = 10). *p < 0.05 versus ND. ^#^ *p* < 0.05 versus HFD. ^&^ *p* < 0.05 versus HFD + ox‐LDL‐M‐EVs. The measurement data were expressed as mean ± standard deviation. One‐way ANOVA was conducted for comparing data between multiple groups, followed by Tukey's post hoc test

To verify the effect of ox‐LDL‐M‐EVs miR‐19b‐3p on atherosclerosis and VSMCs in mice, we injected PKH26 (red fluorescent)‐labelled M‐EVs into ApoE^−/−^ mice. At 21 days after transfection, immunofluorescence revealed the co‐localization of EVs with VSMCs (Figure S4). Meanwhile, we also labelled the aorta in mice treated with ox‐LDL‐M‐EVs with CD68, CD3 and α‐SMA, respectively. Results demonstrated that CD9 was mainly co‐localized with α‐SMA, indicating that ox‐LDL‐M‐EVs were mainly internalized by VSMCs in the plate, but not macrophages, thereby regulating the occurrence and development of atherosclerosis in regulation of the VSMC function (Figure S5). Immunofluorescence further revealed that the expression of α‐SMA and CD9 in HFD‐induced ApoE^−/−^ mice injected with ox‐LDL‐M‐EVs was significantly increased (Figure 6A). RT‐qPCR revealed a high expression pattern of miR‐19b‐3p in the aorta of HFD‐induced ApoE^−/−^ mice injected with ox‐LDL‐M‐EVs (Figure 6B). On the basis of the results from immunochemistry and western blot assay, the expression pattern of JAZF1 in HFD + HFD‐induced ApoE^−/−^ mice injected with ox‐LDL‐M‐EVs was low (Figure 6C,D). The results of plaque area and lipid deposition revealed a markedly increased area of atherosclerotic lesions in the aorta of HFD‐induced ApoE^−/−^ mice injected with ox‐LDL‐M‐EVs (Figure 6E). Altogether, our findings indicated that ox‐LDL‐M‐EVs carrying miR‐19b‐3p can facilitate the progression of atherosclerosis in ApoE^−/−^ mice.

Additionally, we also isolated mouse peritoneal primary macrophages (PM), and separated and purified PM‐EVs and ox‐LDL‐PM‐EVs. As revealed by RT‐qPCR, ox‐LDL treatment markedly increased the expression pattern of miR‐19b‐3p in primary macrophages‐derived EVs (Figure S6A). To further verify the effect of ox‐LDL‐PM‐EVs containing miR‐19b‐3p on atherosclerosis in mice, the ApoE^−/−^ mice were injected with EVs from peritoneal macrophages and detected the plaque area and lipid deposition 21 days after the injection and transfection. Results showed an increased concentration of atherosclerotic lesions in the aorta of HFD mice treated with ox‐LDL‐PM‐EVs (Figure S6B). Coherently, ox‐LDL‐PM‐EVs carrying miR‐19b‐3p can accelerate the progression of atherosclerosis in ApoE^−/−^ mice.

## 4 DISCUSSION

Atherosclerosis is regarded as a chief contributor to cardiovascular disease and Peripheral artery disease.^15^ In the current study, we explored the role of M‐EVs containing miR‐19b‐3p in atherosclerosis and identified that M‐EVs containing miR‐19b‐3p elicited stimulative functions on atherosclerosis development through inhibition of JAZF1 by facilitating the migration and proliferation of VSMCs.

Initially, our findings revealed that the concentration of M‐EVs was increased in atherosclerotic plaques where M‐EVs facilitated the migration and proliferation of VSMCs. Notably, accumulating evidence has identified the roles of macrophages and EVs in the progression of atherosclerosis. For instance, macrophages are considered to be capable of dominating atherosclerosis.^16^ Moreover, increased circulating levels of EVs have been previously observed in patients with cardiovascular disease patients where EVs could serve as modulators of vascular cell functions in atherosclerosis.^17^ Exosomes, a type of EVs, have elicited potential as promising biomarkers in the development of atherosclerosis.^18^ Interestingly, an existing study determined the ability of macrophage foam cell‐derived EVs to radically could contribute to the enhanced migration and adhesion of VSMCs.^19^ Altogether, the aforementioned literature is in consistency with our finding revealed the involvement of M‐EVs are involved in the development of atherosclerosis.

Furthermore, our results identified a high expression pattern of miR‐19b‐3p in the experimental M‐EVs. In consistency with this finding, previous literature has determined the stimulated role of miR‐19b‐3p in atherosclerosis and other cardiovascular diseases. For instance, miR‐19b could progress in dysfunction of endothelial cells to subsequently facilitate the development of atherosclerosis.^11^ Besides, an existing study elicited the ability of an increased plasma expression of miR‐19b‐3p in the early stage of acute myocardial infarction to serve as potential biomarker for the diagnosis of myocardial infarction.^20^ Mechanistically, we found in the current study that miR‐19b‐3p inhibited JAZF1 to promote VSMC migration and proliferation. It is noteworthy that the regulation relationship between miR‐19b‐3p and JAZF1 has been rarely reported. In this study, our dual‐luciferase gene reporter assay confirmed that miR‐19b‐3p could targeted and downregulate JAZF1 in VSMCs during the development of atherosclerosis. Intriguingly, several previous studies have shown the association of JAZF1 with atherosclerosis development and with the cell cycle of VSMCs. For instance, it was found that upregulated JAZF1 could suppress the progression of atherosclerosis in ApoE‐deficient mice via repression of hepatic cholesterol synthesis with the involvement of CREB.^14^ Additionally, JAZF1 exerts important mediatory functions on JNK and p38 MAPK,^21^ which are believed to participate in the regulation of migration and proliferation of VSMCs.^22^ These results revealed that M‐EVs containing miR‐19b‐3p can essentially regulate the migration and proliferation of VSMCs to inherently facilitate the development of atherosclerosis.

Importantly, we confirmed that M‐EVs could transfer miR‐19b‐3p to VSMCs to regulate the progression of VSMCs. Accumulating evidence has determined the regulatory effect of miR‐containing M‐EVs on multiple diseases. For instance, atherogenic M‐EVs carrying miR‐146a w could evidently facilitate the progression of atherosclerosis by hindering cell migration and enhancing the entrapment of macrophages in the vessel wall.^3^ Additionally, an existing study identified that M‐EVs carrying miR by inducing pulmonary smooth muscle proliferation were involved in PAH progression and regulating smooth muscle hyperplasia.^23^, ^24^ Notably, EVs containing miR‐19b‐3p have also been identified to function as an inducer for disease development. For instance, Rodosthenous et al. identified that EVs containing miR‐19b‐3p was partly responsible for the increased long‐term ambient PM2.5 levels and was thus implicated for association with PM‐related cardiovascular disease including atherosclerosis.^12^ Moreover, it has been previously reported that tumor‐derived EVs intrinsically facilitated the proliferation of lung bronchial cells via miR‐19b.^25^ Therefore, it was concluded in the present study that M‐EVs containing miR‐19b‐3p can regulate the migration and proliferation of VSMCs to favour the development of atherosclerosis.

**FIGURE 7:**
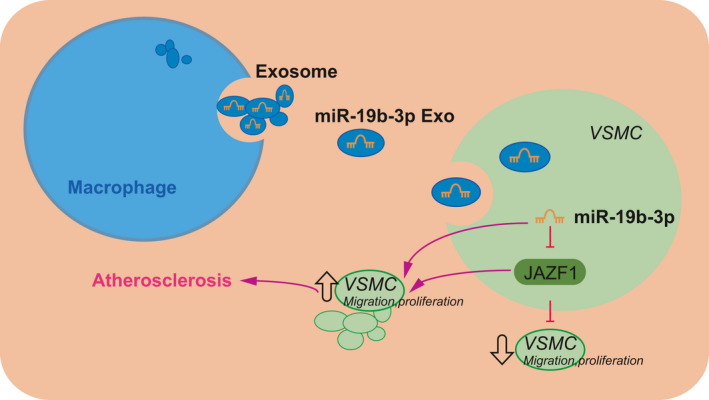
Molecular mechanism of M‐EVs carrying miR‐19b‐3p in atherosclerosis. Macrophages secrete EVs, which transfer miR‐19b‐3p into VSMCs; miR‐19b‐3p inhibits the expression of JAZF1, which promotes the migration and proliferation of VSMCs, thereby participating in the formation of atherosclerosis

To conclude, the results from the current study demonstrate that macrophages can secrete EVs and the M‐EVs transfer miR‐19b‐3p into VSMCs; miR‐19b‐3p targets and inhibits the expression of JAZF1 to facilitate the migration and proliferation of VSMCs, which further facilitates the development of atherosclerosis (Figure 7). Our findings provide an insight for the development of M‐EVs‐based therapy for atherosclerosis, however, the validity of these results in the clinical setting is necessitated. Moreover, the specific mechanisms regarding the involvement of miR‐19b‐3p and JAZF1 in atherosclerosis warrant further examination. Besides, considering that the transition of VSMC phenotype has important pathophysiological significance in different stages of atherosclerosis, we would observe the phenotypic changes in treated VSMCs in future studies.

## CONFLICT OF INTEREST

None.

## AUTHOR CONTRIBUTION

**Qingshan Wang:** Conceptualization (lead); Data curation (lead); Formal analysis (lead); Writing‐original draft (lead); Writing‐review & editing (equal). **Yuandi Dong:** Investigation (lead); Methodology (lead); Project administration (lead); Writing‐review & editing (equal). **Haiyang Wang:** Supervision (lead); Validation (lead); Visualization (lead); Writing‐original draft (supporting).

## Supporting information

Fig S1

Fig S2

Fig S3

Fig S4

Fig S5

Fig S6

Table S1

## Data Availability

Data available on request from the authors.

## References

[1] Libby P , Ridker PM , Hansson GK . Progress and challenges in translating the biology of atherosclerosis. Nature. 2011;473:317‐325. 21593864 10.1038/nature10146

[2] Rodrigues DA , Martins J , e Silva K , et al. GSTM1 polymorphism in patients with clinical manifestations of atherosclerosis. Genet Mol Res. 2017;16. 10.4238/gmr16019101 28362975

[3] Elkind MS . Infectious burden: a new risk factor and treatment target for atherosclerosis. Infect Disord Drug Targets. 2010;10:84‐90. 20166973 10.2174/187152610790963519 PMC2891124

[4] Yoo AR , Koh SH , Cho GW , Kim SH . Inhibitory effects of cilostazol on proliferation of vascular smooth muscle cells (VSMCs) through suppression of the ERK1/2 pathway. J Atheroscler Thromb. 2010;17:1009‐1018. 20720374 10.5551/jat.4309

[5] Gangoda L , Boukouris S , Liem M , Kalra H , Mathivanan S . Extracellular vesicles including exosomes are mediators of signal transduction: are they protective or pathogenic? Proteomics. 2015;15:260‐271. 25307053 10.1002/pmic.201400234 PMC4419270

[6] Lanyu Z , Feilong H . Emerging role of extracellular vesicles in lung injury and inflammation. Biomed Pharmacother. 2019;113:108748. 30877881 10.1016/j.biopha.2019.108748

[7] Moore KJ , Tabas I . Macrophages in the pathogenesis of atherosclerosis. Cell. 2011;145:341‐355. 21529710 10.1016/j.cell.2011.04.005 PMC3111065

[8] Fujita Y , Yoshioka Y , Ito S , Araya J , Kuwano K , Ochiya T . Intercellular communication by extracellular vesicles and their microRNAs in asthma. Clin Ther. 2014;36:873‐881. 24909737 10.1016/j.clinthera.2014.05.006

[9] Ponomarev ED , Veremeyko T , Weiner HL . MicroRNAs are universal regulators of differentiation, activation, and polarization of microglia and macrophages in normal and diseased CNS. Glia. 2013;61:91‐103. 22653784 10.1002/glia.22363 PMC3434289

[10] Laffont B , Rayner KJ . MicroRNAs in the pathobiology and therapy of atherosclerosis. Can J Cardiol. 2017;33:313‐324. 28232017 10.1016/j.cjca.2017.01.001 PMC5421617

[11] Xue Y , Wei Z , Ding H , et al. MicroRNA‐19b/221/222 induces endothelial cell dysfunction via suppression of PGC‐1alpha in the progression of atherosclerosis. Atherosclerosis. 2015;241:671‐681. 26117405 10.1016/j.atherosclerosis.2015.06.031

[12] Rodosthenous RS , Coull BA , Lu Q , Vokonas PS , Schwartz JD , Baccarelli AA . Ambient particulate matter and microRNAs in extracellular vesicles: a pilot study of older individuals. Part Fibre Toxicol. 2016;13:13. 26956024 10.1186/s12989-016-0121-0 PMC4782360

[13] Bae KB , Kim MO , Yu DH , et al. Overexpression of Jazf1 induces cardiac malformation through the upregulation of pro‐apoptotic genes in mice. Transgenic Res. 2011;20:1019‐1031. 21221781 10.1007/s11248-010-9476-4

[14] Li X , Yang M , Wang H , et al. Overexpression of JAZF1 protected ApoE‐deficient mice from atherosclerosis by inhibiting hepatic cholesterol synthesis via CREB‐dependent mechanisms. Int J Cardiol. 2014;177:100‐110. 25499349 10.1016/j.ijcard.2014.09.007

[15] Segedy AK , Pyle AL , Li B , et al. Identification of small proline‐rich repeat protein 3 as a novel atheroprotective factor that promotes adaptive Akt signaling in vascular smooth muscle cells. Arterioscler Thromb Vasc Biol. 2014;34:2527‐2536. 25278290 10.1161/ATVBAHA.114.303644 PMC4239161

[16] Gronholdt ML , Nordestgaard BG , Bentzon J , et al. Macrophages are associated with lipid‐rich carotid artery plaques, echolucency on B‐mode imaging, and elevated plasma lipid levels. J Vasc Surg. 2002;35:137‐145. 11802145

[17] van der Vorst EPC , de Jong RJ , Donners M . Message in a microbottle: modulation of vascular inflammation and atherosclerosis by extracellular vesicles. Front Cardiovasc Med. 2018;5:2. 29404342 10.3389/fcvm.2018.00002 PMC5786527

[18] Lu M , Yuan S , Li S , Li L , Liu M , Wan S . The exosome‐derived biomarker in atherosclerosis and its clinical application. J Cardiovasc Transl Res. 2019;12:68‐74. 29802541 10.1007/s12265-018-9796-y

[19] Niu C , Wang XU , Zhao M , et al. Macrophage foam cell‐derived extracellular vesicles promote vascular smooth muscle cell migration and adhesion. J Am Heart Assoc. 2016;5. 10.1161/JAHA.116.004099 PMC5121506 27792649

[20] Wang K‐J , Zhao X , Liu Y‐Z , et al. Circulating MiR‐19b‐3p, MiR‐134‐5p and MiR‐186‐5p are promising novel biomarkers for early diagnosis of acute myocardial infarction. Cell Physiol Biochem. 2016;38:1015‐1029. 26939053 10.1159/000443053

[21] Yang M , Dai J , Jia Y , et al. Overexpression of juxtaposed with another zinc finger gene 1 reduces proinflammatory cytokine release via inhibition of stress‐activated protein kinases and nuclear factor‐kappaB. FEBS J. 2014;281:3193‐3205. 24854865 10.1111/febs.12853

[22] Zhan Y , Kim S , Izumi Y , et al. Role of JNK, p38, and ERK in platelet‐derived growth factor‐induced vascular proliferation, migration, and gene expression. Arterioscler Thromb Vasc Biol. 2003;23:795‐801. 12637337 10.1161/01.ATV.0000066132.32063.F2

[23] Nguyen M‐A , Karunakaran D , Geoffrion M , et al. Extracellular vesicles secreted by atherogenic macrophages transfer microRNA to inhibit cell migration. Arterioscler Thromb Vasc Biol. 2018;38:49‐63. 28882869 10.1161/ATVBAHA.117.309795 PMC5884694

[24] Sharma H , Chinnappan M , Agarwal S , et al. Macrophage‐derived extracellular vesicles mediate smooth muscle hyperplasia: role of altered miRNA cargo in response to HIV infection and substance abuse. FASEB J. 2018;32:5174‐5185. 29672222 10.1096/fj.201701558R PMC6103174

[25] Borzi C , Calzolari L , Ferretti AM , et al. c‐Myc shuttled by tumour‐derived extracellular vesicles promotes lung bronchial cell proliferation through miR‐19b and miR‐92a. Cell Death Dis. 2019;10:759. 31591389 10.1038/s41419-019-2003-5 PMC6779734

