# Supplementary material for: microRNA‐19b‐3p‐containing extracellular vesicles derived from macrophages promote the development of atherosclerosis by targeting JAZF1
Source: J Cell Mol Med. 2021 Dec 14;26(1):48–59. doi: 10.1111/jcmm.16938 (PMC8742201; doi:10.1111/jcmm.16938)
Supplement: Supplementary file 1 — Fig S1 [file JCMM-26-48-s007.docx]

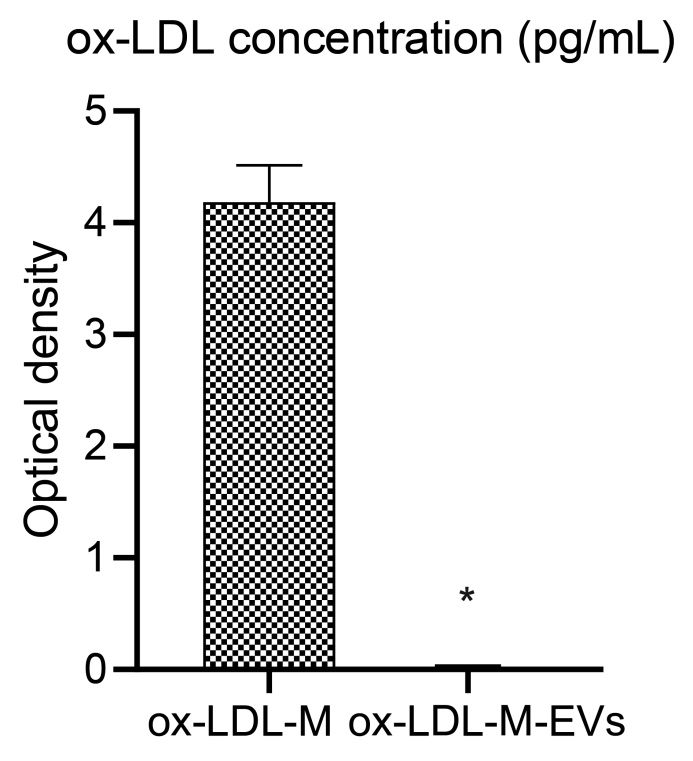


**Figure S1** ox-LDL content. The content of ox-LDL (pg/mL) in oe-LDL-M and ox-LDL-M-EVs measured with ELISA, * *p* < 0.05. *vs.* oe-LDL-M. The measurement data were expressed as mean ± standard deviation. Unpaired *t*-test was used for comparing data between two groups.
