# Supplementary material for: microRNA‐19b‐3p‐containing extracellular vesicles derived from macrophages promote the development of atherosclerosis by targeting JAZF1
Source: J Cell Mol Med. 2021 Dec 14;26(1):48–59. doi: 10.1111/jcmm.16938 (PMC8742201; doi:10.1111/jcmm.16938)
Supplement: Supplementary file 2 — Fig S2 [file JCMM-26-48-s002.docx]

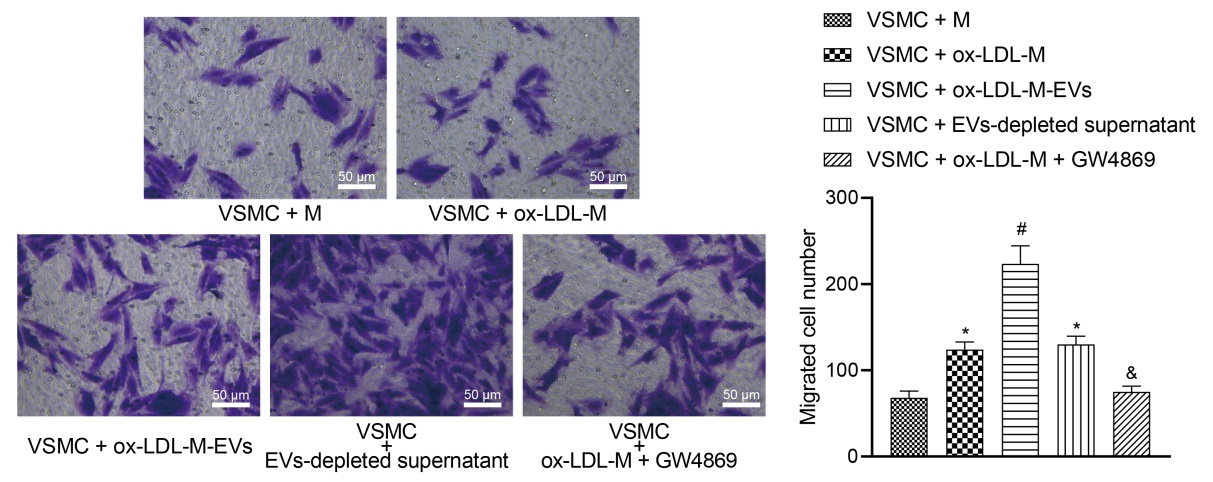


**Figure S2** The migration ability of VSMCs. The migration ability of VSMCs in response to Control, ox-LDL-M-EVs-miR-NC, ox-LDL-M-EVs-miR-mimic, ox-LDL-M-EVs-KD-NC, or ox-LDL-M-EVs-KD measured with Transwell. * *p* < 0.05. *vs.* control. ^#^ *p* < 0.05. *vs.* ox-LDL-M-EVs-miR-NC. ^&^ *p* < 0.05. *vs.* ox-LDL-M-EVs-KD-NC. The measurement data were expressed as mean ± standard deviation. One-way ANOVA was conducted for comparing data between multiple groups, followed by Tukey’s post hoc test.
