# Supplementary material for: microRNA‐19b‐3p‐containing extracellular vesicles derived from macrophages promote the development of atherosclerosis by targeting JAZF1
Source: J Cell Mol Med. 2021 Dec 14;26(1):48–59. doi: 10.1111/jcmm.16938 (PMC8742201; doi:10.1111/jcmm.16938)
Supplement: Supplementary file 3 — Fig S3 [file JCMM-26-48-s004.docx]

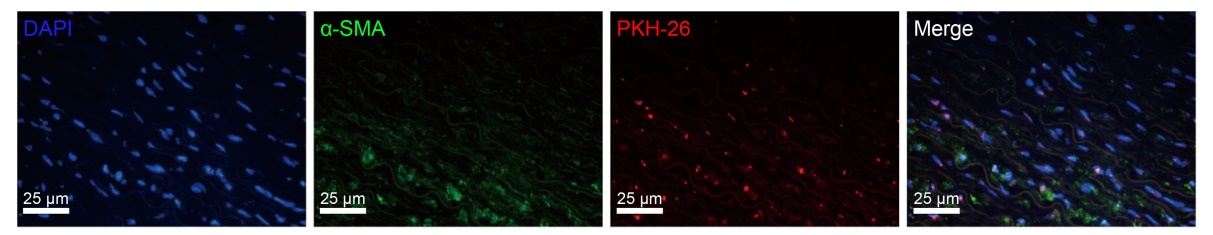


**Figure S3** ox-LDL-PM-EVs regulate JAZF1 by delivery of miR-19b-3p. JAZF1 expression in VSMCs determined with Western blot assay. * *p* < 0.05. *vs.* ox-LDL-M. ^#^ *p* < 0.05. *vs.* ox-LDL-M-EVs-miR-NC. ^&^ *p* < 0.05. *vs.* ox-LDL-M-EVs-KD-NC. The measurement data were expressed as mean ± standard deviation. One-way ANOVA was conducted for comparing data between multiple groups, followed by Tukey’s post hoc test.
