# Supplementary material for: microRNA‐19b‐3p‐containing extracellular vesicles derived from macrophages promote the development of atherosclerosis by targeting JAZF1
Source: J Cell Mol Med. 2021 Dec 14;26(1):48–59. doi: 10.1111/jcmm.16938 (PMC8742201; doi:10.1111/jcmm.16938)
Supplement: Supplementary file 4 — Fig S4 [file JCMM-26-48-s003.docx]

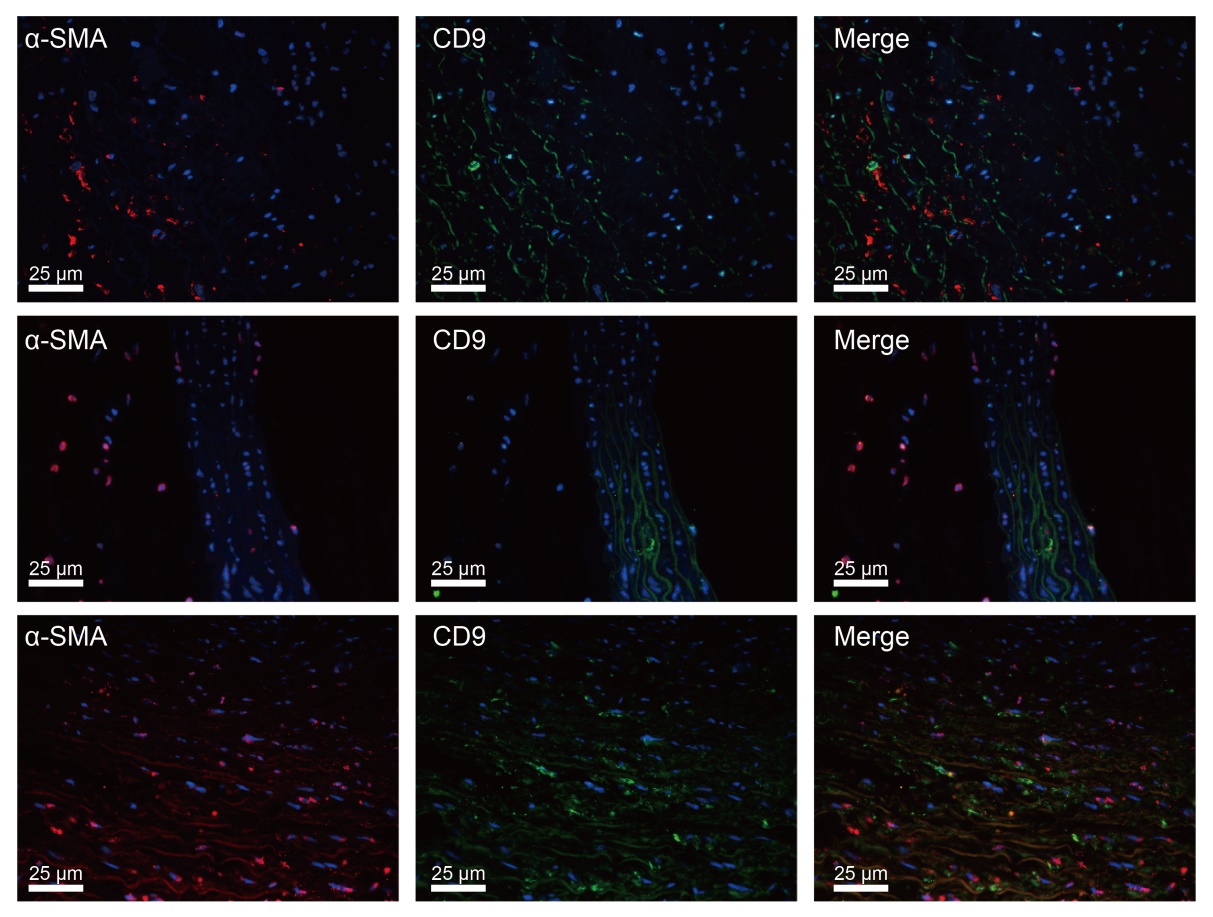


**Figure S4** The co-localization of PKH26 labeled M-EVs and VSMCs. The co-localization of PKH26 (red fluorescence) labeled M-EVs and α-SMA (green fluorescence) determined with Immunofluorescence.
