# Supplementary material for: microRNA‐19b‐3p‐containing extracellular vesicles derived from macrophages promote the development of atherosclerosis by targeting JAZF1
Source: J Cell Mol Med. 2021 Dec 14;26(1):48–59. doi: 10.1111/jcmm.16938 (PMC8742201; doi:10.1111/jcmm.16938)
Supplement: Supplementary file 5 — Fig S5 [file JCMM-26-48-s001.docx]

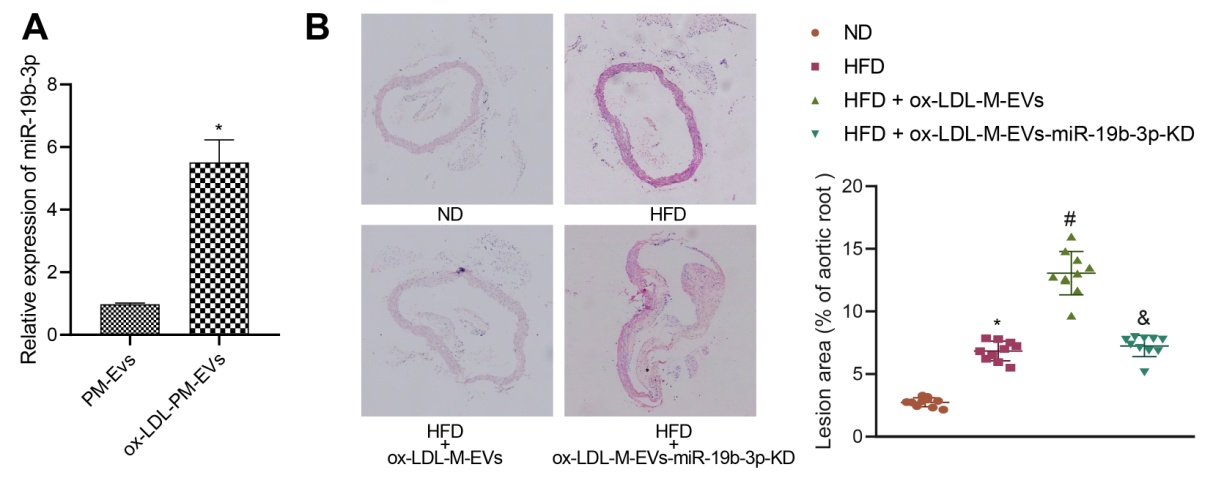


**Figure S5** The co-localization of CD68, CD31, and α-SMA with CD9. The co-localization of CD68, CD31, and α-SMA with CD9 determined with Immunofluorescence.
