# Supplementary material for: microRNA‐19b‐3p‐containing extracellular vesicles derived from macrophages promote the development of atherosclerosis by targeting JAZF1
Source: J Cell Mol Med. 2021 Dec 14;26(1):48–59. doi: 10.1111/jcmm.16938 (PMC8742201; doi:10.1111/jcmm.16938)
Supplement: Supplementary file 6 — Fig S6 [file JCMM-26-48-s006.docx]

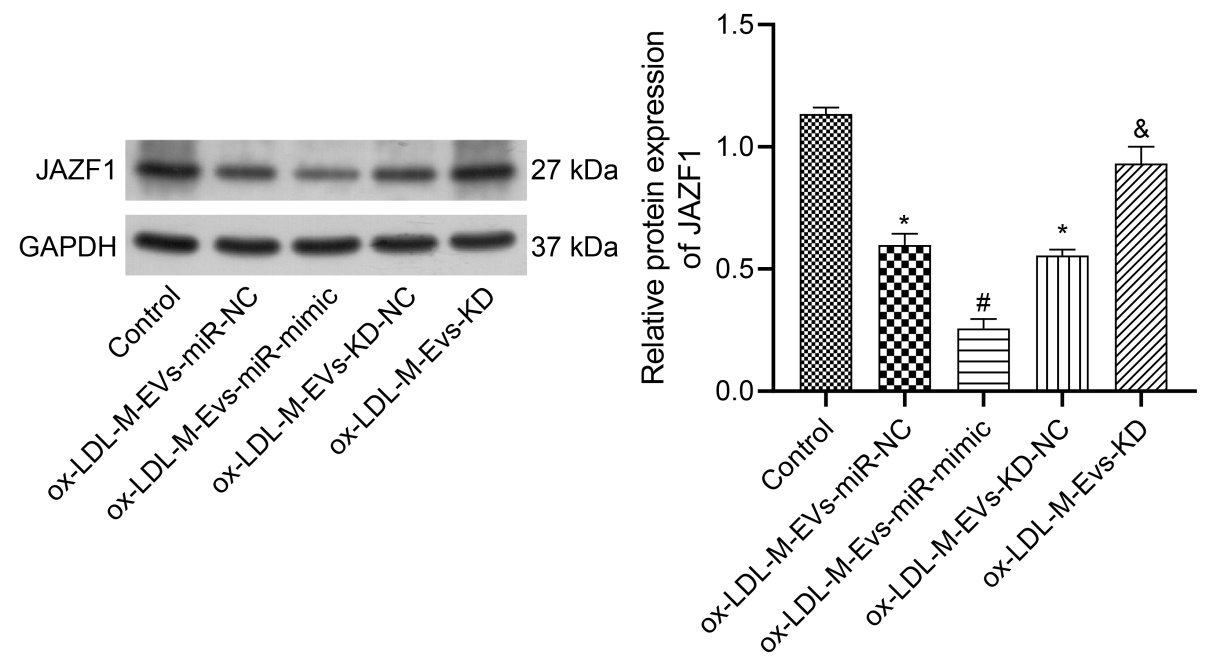


**Figure S6** Purification of PM-EVs and ox-LDL-PM-EVs and injection into ApoE^-/-^ mice.A, The expression of miR-19b-3p in primary macrophages-derived EVs and ox-LDL-PM-EVs. B, HE staining of the plaque area and lipid deposition 21 days after the injection and transfection (n = 10). * *p* < 0.05. *vs.* ND mice. ^#^ *p* < 0.05. *vs.* HFD mice. ^&^ *p* < 0.05. *vs.* HFD+ox-LDL-M-EVs. The measurement data were expressed as mean ± standard deviation. Unpaired *t*-test was used for comparing data between two groups. One-way ANOVA was conducted for comparing data between multiple groups, followed by Tukey’s post hoc test.
