# Supplementary material for: microRNA‐19b‐3p‐containing extracellular vesicles derived from macrophages promote the development of atherosclerosis by targeting JAZF1
Source: J Cell Mol Med. 2021 Dec 14;26(1):48–59. doi: 10.1111/jcmm.16938 (PMC8742201; doi:10.1111/jcmm.16938)
Supplement: Supplementary file 7 — Table S1 [file JCMM-26-48-s005.docx]

**Table S1** Primer sequences for RT-qPCR

| Gene | Sequences |
| --- | --- |
| miR-19b-3p | F: 5-AACAGAAGTTTTGCAGGTTTGCATC-3 |
|  | R: 5-CAGTGCAGGGTCCGAGGT-3 |
| JAZF1 | F: 5-ACGCCGAGAACAGGAAT-3 |
|  | R: 5-GTGCTGCTGCGGAATGAA-3 |
| β-actin | F: 5-ACCACAGCTGAGAGGGAAATCG-3 |
|  | R: 5-AGAGGTCTTTACGGATGTCAACG-3 |
| U6 | F: 5-GCTTCGGCAGCACATATACTAAAAT-3 |
|  | R: 5-CGCTTCACGAATTTGCGTGTCAT-3 |

Note: miR-19b-3p, microRNA-19b-3p; JAZF1, Juxtaposed with another zinc finger gene 1; RT-qPCR, reverse transcription-quantitative polymerase chain reaction
